# Antioxidant and Anti-Inflammatory Activities of *Kigelia africana* (Lam.) Benth.

**DOI:** 10.1155/2020/4352084

**Published:** 2020-06-17

**Authors:** Alice Nabatanzi, Sanah M. Nkadimeng, Namrita Lall, John D. Kabasa, Lyndy J. McGaw

**Affiliations:** ^1^Department of Plant Sciences, Microbiology and Biotechnology, College of Natural Sciences, Makerere University, Kampala 00256, Uganda; ^2^Phytomedicine Programme, Department of Paraclinical Sciences, Faculty of Veterinary Science, University of Pretoria, Onderstepoort 0110, South Africa; ^3^Department of Plant and Soil Sciences, University of Pretoria, Hatfield 0028, South Africa; ^4^College of Veterinary Medicine, Animal Resources and Biosecurity, Makerere University, Kampala 00256, Uganda; ^5^Future Africa, University of Pretoria, Hatfield 0028, South Africa; ^6^School of Natural Resources, University of Missouri, Columbia 65211, USA; ^7^College of Pharmacy, JSS Academy of Higher Education and Research, Mysuru, Karnataka 570015, India

## Abstract

*Kigelia africana* is used to manage inflammation among indigenous communities. We hypothesized that *K. africana* extracts contain phytoconstituents with good antioxidant and anti-inflammatory activities. The methanolic extract of *K. africana* fruits and *Spathodea campanulata* leaves (SPK04), *K. africana* aqueous fruit extract (KFM02), and *K. africana* acetone fruit extract (KFM05) were subjected to antioxidant and anti-inflammatory assays. Antioxidant activity was evaluated using the ABTS radical scavenging assay, and the MTT cell viability assay was used for cytotoxicity. The extracts were preincubated with enzymes and assayed for 15-LOX and COX-2 enzyme activity using an ELISA method. Nitric oxide (NO) inhibitory effect of the extracts was evaluated and measurement of proinflammatory cytokines (IL-1*β*, TNF-*α*, and IL-6) and the anti-inflammatory cytokine (IL-10) was done using ELISA kits. SPK04 had the highest antioxidant activity with a mean inhibition of 99.37 ± 0.56% and an IC_50_ of 4.28 *µ*g/mL. SPK04 and KFM05 did not inhibit 15-LOX as their IC_50_ values were >1000 *μ*g/mL. All extracts were safe on Vero cells at the highest concentration (200 *µ*g/mL) tested. KFM02 was the best inhibitor of NO production and had the highest cell viability at both the lowest (50 *µ*g/mL) and highest concentrations (200 *µ*g/mL). SPK04 was the best COX-2 inhibitor while KFM05 expressed the strongest suppression effect for IL-*β* and IL-6. KFM02 did not inhibit IL-6 at the highest concentration (200 *µ*g/mL). The order of suppression of TNF-*α* by the extracts differed across concentrations, KFM05 > SPK04 > KFM02 at 200 *µ*g/mL, KFM02 > SPK04 > KFM05 at 100 *µ*g/mL, and SPK04 > KFM02 > KFM05 at 50 *µ*g/mL. All the tested extracts had no inhibitory effect against IL-10. SPK04, KFM05, and KFM02 had good antioxidant and anti-inflammatory activity and this supports their use as potential anti-inflammatory therapies. This study presents for the first time the antioxidant and anti-inflammatory activity of *K. africana* and *S. campanulata* polyherbal extract. It is also among the very few studies that have reported the inhibitory effect of cytokines IL-1*β*, TNF-*α*, IL-6, and IL-10 by *K. africana*.

## 1. Introduction

Inflammation is the body's first protective mechanism to enable tissue healing when faced with injurious stimuli [1]. The body responds to inflammation by releasing proinflammatory cytokines such as interleukins (IL-1*β* and IL-6), interferons (IFN), and tumor necrosis factor-alpha (TNF-*α*) among others [2]. This is followed by induction of cyclooxygenase-2 (COX-2) which then synthesizes prostaglandins (PGs) that contribute to inflammation, swelling, and pain [3]. Lipoxygenase (LOX) metabolises arachidonic acids to leukotrienes, a group of inflammatory mediators [4]. Furthermore, when macrophages are activated, they produce reactive oxygen species (ROS) which cause oxidative stress (OS). Oxidative stress is an inflammatory mediator which stimulates the release of proinflammatory cytokines and nitric oxide (NO) [5]. Nitric oxide contributes to the exacerbation of inflammatory diseases, inhibits mitochondrial enzymes, and activates COXs to produce PGs [6]. Therefore, an inhibitor of COX-2, LOX, NO, and ROS is likely to be effective in preventing and treating inflammatory diseases. The conventional treatment for inflammation is the use of nonsteroidal anti-inflammatory drugs (NSAIDs) [7] among others, but prolonged use of such substances results in toxic effects. A variety of plant constituents such as coumarins, carotenoids, and flavonoids [8] have been isolated and shown to have anti-inflammatory activities both *in vitro* and *in vivo*. Hence, the treatment of inflammatory diseases by herbal drugs has been of interest to researchers.

Several scholars have reported *K. africana*'s traditional uses as an anti-inflammatory among other uses in various communities. *K. africana* fruits boiled with milk are used in Botswana to treat sexually transmitted diseases; the bark of *K. africana* is used in Nigeria to treat inflammation, dysentery, and cancer [9, 10], and *K. africana* fruits are used as anti-inflammatories in Kenya, Embu community [11]. Furthermore, antioxidant [12–14] and anti-inflammatory studies [15, 16] have been conducted on the various parts of *K. africana* and the species has proven effective. The anti-inflammatory activity of *S. campanulata* has also been documented and scientifically validated. In Senegal, bruised leaves and flowers of *S. campanulata* are applied to wounds [17] as a poultice. The methanol and aqueous extracts of *S. campanulata* leaves have been proven to have anti-inflammatory (paw edema induced by carrageenan) activity in rats [18]. Traditionally, *K. africana* has been used either as a single plant or in combination with other plants for increased potency. In Africa, *K. africana*, *Hypoxis hemerocallidea*, and *Senecio serratuloides* leaves and roots decoction is used to treat sexually transmitted infections and sores [19]. In Uganda, *S. campanulata* bark mixed with *K. africana* fruits is used as a dressing for wounds and in the treatment of various skin diseases. Therefore, *S. campanulata*'s anti-inflammatory activity has been justified both traditionally and scientifically more reason for its adoption as a polyherbal with *K. africana* in the current study.

The anti-inflammatory activity of *K. africana* has been proposed to act by suppressing inflammatory mediators [20]. The present study ascertained the anti-inflammatory activity of *K. africana* extracts through the inhibition of ROS (antioxidant activity), 15-LOX, NO, COX-2, proinflammatory cytokines, and anti-inflammatory cytokines to enhance understanding of the possible mechanisms of activity against inflammation. The cytotoxicity on normal mammalian (Vero African green monkey kidney) cells was also established.

## 2. Methods

### 2.1. Plant Collection

*Kigelia africana* (leaves and fruits) and *Spathodea campanulata* Beauv. (leaves) were collected in the summer of April 2019 from Lynnwood, Pretoria East, South Africa. Voucher specimens for each of the plant species were prepared and deposited at the HGWJ Schweickerdt Herbarium, University of Pretoria. Herbarium voucher specimen numbers PRU/1/125491/Nabatanzi SA for *K. africana* and PRU/1/125492002/Nabatanzi SA for *S. campanulata* were assigned.

### 2.2. Plant Storage

The leaves of the two plant species were separated from their stems, cleaned of any extraneous matter, and dried separately for 14 days at room temperature. The fruits were washed with running tap water, cut into small pieces, and oven-dried at 40°C for 16 days. The dried plants were milled to a fine powder in a Macsalab mill (Model 200 LAB; Eriez, Bramley, South Africa) and stored at room temperature in closed containers in the dark until used.

### 2.3. Extraction Procedure

Extraction was done at a ratio of 1 g of finely ground plant material to 10 mL of solvent. Extracts were then prepared:

  Extract 1 (SPK04): equal amounts of *K. africana* leaves (250 g) and *S. campanulata* leaves (250 g) were weighed into one big glass container and to it 5 L of methanol was added
  Extract 2 (KFM02): 500 g of *K. africana* fruits was weighed into a big glass container and to it 5 L of hot water was added
  Extract 3 (KFM05): 500 g of *K. africana fruits* was weighed into a big glass container and to it 5 L of acetone was added

All the solvents used were of technical grade (Merck, Johannesburg, South Africa). After adding the solvents to the powdered samples, the containers and contents were vigorously shaken for 20 minutes on a Labotec model 20.2 shaking machine at high speed. The containers of extracts were then covered with silver foil and macerated for 24 hours at room temperature. After 24 hours, the particulate matter of each extract had sedimented and the supernatant portion was filtered with 0.1 mm^2^ mesh gauze and then with Whatman No 1 filter paper with a pore size of 11 *μ*m. The water extract (KFM02) was frozen at −40°C and lyophilized in a VIRTIS Benchtop 2 K, 4 K, and 6 K Freeze Dryer, United Scientific. The methanol (SPK04) and acetone (KFM05) extracts were evaporated using a rotavapor (R-114; Büchi, Newcastle, USA) and decanted into preweighed labelled beakers. The extracts were then placed under a stream of air in a fume cupboard at room temperature for further drying. On complete drying, they were stored at refrigerator temperature (2–6°C) until used.

### 2.4. Antioxidant Activity: 2,2′-Azino-bis (3-Ethylbenzthiazoline-6-sulfonic Acid (ABTS) Radical Scavenging Assay

The ABTS radical scavenging capacity of the samples was measured as described by Re et al. [21]. Ascorbic acid was used as a positive control and the extract without ABTS as blank. ABTS^+^ was generated by reacting 7 mM ABTS aqueous solution with 2.4 mM potassium persulfate in the dark for 16 hours at room temperature. Prior to assay, this solution was diluted in methanol and equilibrated at 30°C to give an absorbance of 0.7 ± 0.02 at 734 nm (BioTek Synergy microplate reader). Ten microlitres of each extract at different concentrations was added to 1 mL of diluted ABTS solution in U-bottom-shaped 96-well microplates. The plates were incubated for 5 min at 37°C under dark conditions, then the absorbance was measured at 734 nm, and the percentage of inhibition was calculated. The percentage ABTS inhibition was calculated using the following formula:

(1) $$\begin{array}{c} \text{scavenging capacity }\%=100-\frac{\text{OD sample}*\text{OD sample blank}}{\text{OD control}*\text{OD control blank}}\times 100\%, \end{array}$$

where OD is the absorbance.

The IC_50_ values were calculated from the graph plotted as inhibition percentage against the concentration.

### 2.5. Inhibition of 15-Lipoxygenase (15-LOX) Enzyme

The lipoxygenase inhibitor screening assay kit [22] was used to screen for inhibitors of 15-LOX. The 15-LOX (Sigma) was made up to a working solution of 200 units/mL and kept on ice. A volume of 12.5 *μ*L of test sample or control (dissolved in DMSO) was added to 487.5 *μ*L of 15-LOX in a 96-well microtitre plate and incubated at room temperature for 5 min. After incubation, 500 *μ*L of substrate solutions (10 *μ*L linoleic acid dissolved in 30 *μ*L ethanol, made up to 120 mL with 2 M borate buffer at pH 9.0) was added to the solution. After 5 min incubation at room temperature, the absorbance was measured at 234 nm. Quercetin (1 mg/mL) was used as a positive control, while DMSO was used as the negative control (100% enzyme activity or no enzyme inhibition). The percentage enzyme inhibition of each extract compared with negative control as 100% enzyme activity was calculated using the following equation:

(2) $$\begin{array}{c} \text{percent inhibition }\left(\%\right)=\frac{\left(\text{activity of control}-\text{activity of test}\right)}{\text{activity of control }}\times \;100. \end{array}$$

The results were expressed as IC_50_, i.e., concentration of the extracts and controls that resulted in 50% 15-LOX inhibition plotted on a graph.

### 2.6. The 3-(4,5-Dimethylthiazol-2-yl)-2,5-diphenyl Tetrazolium Bromide (MTT) Cell Viability Assay

Cytotoxicity of each extract at different concentrations against Vero monkey kidney cells was determined as described by Mosmann [23]. Cells were seeded at a density of 1 × 10^5^ cells/mL (100 *μ*L) in 96-well microtitre plates and incubated at 37°C and 5% CO_2_ in a humidified environment. After 24-hour incubation, extracts (100 *μ*L) at varying final concentrations were added to the wells containing cells. Doxorubicin (0.38–40 *μ*M) was used as a positive control. A suitable blank control with equivalent volume of acetone was also included, and the plates were further incubated at 3°C for 48 hours in a CO_2_ incubator. The medium was removed by aspiration, and cells were then washed twice with phosphate-buffered saline (PBS), followed by suspension in fresh medium (200 *μ*L). Then, 30 *μ*L of MTT (5 mg/mL in PBS) was added to each well and the plates were incubated at 37°C for 4 hours. The medium was removed by aspiration and 100% DMSO (100 *μ*L) was added to dissolve the formed formazan crystals. The absorbance was measured using a microtitre plate reader (BioTek Synergy) at 570 nm. The percentage of cell growth inhibition was calculated based on a comparison with that of untreated cells. The selectivity index (SI) values were calculated by dividing cytotoxicity LC_50_ values by the MIC values (SI = LC_50_/MIC).

### 2.7. Inhibition of Nitric Oxide (NO) Production

#### 2.7.1. Cell Culture

The RAW 264.7 macrophage cell line was obtained from the American Type Culture Collection (Rockville, MD, USA) and cultured in plastic culture flasks in Dulbecco's modified Eagle's medium (DMEM) containing l-glutamine supplemented with 10% foetal calf serum (FCS) and 1% PSF (penicillin/streptomycin/fungizone) solution under 5% CO_2_ at 37°C and was split twice a week.

All cells were seeded in 96-well microtitre plates and activated by incubation in medium containing LPS (5 *μ*g/mL), and various concentrations of extracts were dissolved initially in DMSO and made up to the required concentration in DMEM. Measurement of nitrite released from macrophages was assessed by the determination of nitrite concentration in the culture supernatant using the Griess reagent. After 24-hour incubation, 100 *μ*L of the supernatant from each well of the cell culture plates was transferred into 96-well microtitre plates and an equal volume of Griess reagent (Sigma) added. The absorbance of the resultant solutions in the wells of the microtitre plate was determined after 10 min at 550 nm. The concentrations of nitrite were calculated from regression analysis using serial dilutions of sodium nitrite as a standard. Percentage inhibition was calculated based on the ability of extracts to inhibit nitric oxide formation by cells compared with the control (cells in media without extracts containing triggering agents and DMSO), which was considered as 0% inhibition.

### 2.8. Cyclooxygenase-2 (COX-2) Inhibition Assay

The COX inhibition screening assay directly measures PGF2*α* by stannous chloride reduction of COX-derived PGH2 produced in the COX reaction [24]. The reaction system consists of reaction buffer, haem, enzyme, and plant extract preincubated at 37°C for 20 min with background and enzyme controls. The reaction was initiated with the addition of arachidonic acid and incubated for 2 min at 37°C. The reaction was stopped with the addition of saturated stannous chloride solution for 5 min at room temperature. The prostaglandins were quantified by enzyme immunoassay technique (EIA). An aliquot of these reactions was added to the precoated plates in triplicate together with acetylcholinesterase (AChE) tracer and antiserum and incubated for 18 hours at room temperature on an orbital shaker. The plate was then finally developed with Ellman's reagent and kept on an orbital shaker in the dark at room temperature for 60 minutes. The absorbance was read at 420 nm. The data were plotted as %B/B0 (standard bound/maximum bound) versus log concentration using a 4-parameter logistic curve fit. The concentration of each sample was determined from a standard curve with appropriate dilutions and used to calculate the percent inhibition as per the following formula:

(3) $$\begin{array}{c} \text{percent inhibition }\left(\%\right)=\frac{\left(\text{activity of control}-\text{activity of test}\right)}{\text{activity of control}}\;\times \;100. \end{array}$$

The results were expressed as IC_50_, i.e., concentration of the extracts and controls that resulted in 50% COX-2 inhibition plotted on a graph.

### 2.9. Cytokine Detection

RAW 264.7 macrophages were stimulated with LPS which led to the secretion of IL-1*β*, TNF-*α*, IL-6, and IL-10 cytokines. Treatment with the plant extracts was then applied, and subsequently, the amounts of IL-1*β*, TNF-*α*, IL-6, and IL-10 were measured using commercial ELABSCIENCE ELISA kits: Mouse IL-6 ELISA Kit, Catalogue no: E-EL-M0044; Mouse TNF-*α* ELISA Kit, Catalogue no: E-EL-M0049; Mouse IL-10 ELISA Kit, Catalogue no. E-EL-M0046; and Mouse IL-1*β* ELISA Kit, Catalogue no: E-EL-M0037. The absorbance was read at 450 nm using a microtitre plate reader (BioTek Synergy). The amounts of IL-1*β*, TNF-*α*, IL-6, and IL-10 were calculated with the help of a standard curve, which was constructed using serial dilutions of cytokine standards provided with the kit. By comparing the OD of the samples to the standard curve, the concentration of the cytokines in the culture supernatant was determined.

### 2.10. Statistical Analysis

Results are expressed as mean ± SEM of three independent experiments. Multigroup analysis was performed using one-way analysis of variance (ANOVA). Significance levels were set as *p* < 0.05.

## 3. Results and Discussion

### 3.1. *Kigelia africana* Extracts Are Highly Potent Free Radical Scavengers

The percentage mean antioxidant capacity of the extracts is presented in Table 1, and the mean IC_50_ values are presented in Table 2. Ascorbic acid was used as a standard with a mean antioxidant activity of 103.95 ± 4.09% and 99.97 ± 0.12% at 100 mg/mL and 1.56 mg/mL concentrations, respectively. The extracts scavenged the ABTS radical in a concentration-dependent manner. SPK04 possessed the best antioxidant activity, which was 99.37 ± 0.56% and 18.03 ± 4.25% at the highest and lowest concentrations, respectively. KFM02 showed the least antioxidant activity of 109.12 ± 14.32% and 15.3 ± 0.88% at the highest and lowest concentrations, respectively. If antioxidant IC_50_ values are <50 *µ*g/mL, as was exhibited by SPK04, KFM05, and KFM02 with 4.28, 19.47, and 21.29 *µ*g/mL, respectively (Table 2), it is an indicator that the plant extracts are potent scavengers. Thus, the lower the IC_50_ value, the more potent is the substance at scavenging ABTS and this implies a higher antioxidant activity [25]. The antioxidant ranking order for the three plant extracts tested was SPK04 > KFM05 > KFM02. Kigelinone and kigelinol were proposed to be the phytochemicals mainly responsible for *K. africana*'s antioxidant power [26], although others may perhaps also be present. Additionally, free radical eradication by *K. africana* may be due to the presence of phenols that can donate the hydrogen atoms in their hydroxyl groups [27]. When the amount of ROS produced exceeds the antioxidant capability of the target cell, oxidative stress occurs [28]. Whereas ROS and NO exacerbate inflammation, antioxidants help in scavenging them, thus avoiding cellular injury and diseases [29]. Therefore, the antioxidant effect of *Kigelia* extracts could contribute to reinforcing their anti-inflammatory properties. Researchers have established the link between oxidants, inflammatory reactions, and infection. In chronic infection and inflammation, the release of leukocytes and other phagocytic cells defends the organism from further injury. The cells do this by releasing free oxidant radicals such as NO, O_2_^−^, H_2_O_2_, and OH^−^ whose actions are inhibited by antioxidants [30].

Several researchers have reported the abundance of antioxidant compounds in plants, thereby justifying their role in scavenging free radicals, thus providing protection to humans against oxidative DNA damage [31–34]. *Kigelia africana*'s antioxidant activity has been investigated by earlier researchers and the reported potency concurs with the results in the current study. A study by Hussein et al. [12] reported that the ethanolic bark (67.33%), fruit (62.66%), and leaf (59.66%) extracts of *K. africana* possessed good antioxidant activity compared with the standard (quercetin) (94%) in a DPPH assay. In another study, the methanolic stem bark extract of *K. africana* significantly scavenged DPPH stable radical by 53.2%, compared to the standard ascorbic acid [14]. According to Olubunmi et al. [35], the free radical scavenging activities of *K. africana* root extract through spectrophotometric assay on the reduction of DPPH were compared favourably with *α*-tocopherol (standard) at the highest concentration (1000 *μ*g/mL). Our data, therefore, supported the findings of previous studies who reported strong antioxidant activity by *K. africana.* Therefore, the analysed plant extracts could be developed into natural antioxidants, which have currently attracted considerable attention.

### 3.2. Treatment with *Kigelia africana* Extracts Did Not Suppress 15-Lipoxygenase (15-LOX) Enzyme

The plant extracts reconstituted in DMSO were evaluated for their effect on the activity of 15-LOX, and the results expressed as IC_50_ values are presented in Table 2. SPK04 and KFM05 did not inhibit 15-LOX as their IC_50_ values were >1000 *μ*g/mL. KFM02 slightly inhibited 15-LOX although it was still a very poor inhibitor with an IC_50_ value >307.12 ± 4.68 *μ*g/mL. Compared to the IC_50_ of the positive control quercetin (24.69 ± 1.43 *μ*g/mL), none of the plant extracts inhibited the 15-LOX enzyme. The isomeric enzyme, 15-LOX, plays a role in many inflammatory disorders and is involved in the synthesis of leukotrienes from arachidonic acids. Biologically active leukotrienes are mediators of many proinflammatory and allergic reactions; therefore, the inhibition of the synthesis of leukotrienes by 15-LOX is considered as an important therapeutic strategy [36]. Therefore, the extracts were not able to block the formation of leukotrienes via the arachidonic acid pathway by inhibiting lipoxygenase.

### 3.3. *Kigelia africana* Extracts Are Not Cytotoxic to Vero Monkey Kidney Cells

Before selection of extracts for further anti-inflammatory analyses, their toxicity on normal mammalian cells has to be established. It is of paramount importance that only nontoxic extracts be subjected to further anti-inflammatory analyses. Our results indicated that all the analysed plant extracts were safe on Vero cells at the highest concentration tested when compared with the positive control (doxorubicin) which had an LC_50_ of 0.01 ± 0.00 *μ*g/mL (Table 2). SPK04 was the safest extract, followed by KFM05 and KFM02 with LC_50_ values of 0.32 ± 0.11, 0.26 ± 0.12, and 0.20 ± 0.01 *μ*g/mL, respectively. Doxorubicin is a standard drug commonly used to treat leukaemia and Hodgkin's lymphoma, as well as cancers of the bladder, breast, stomach, lung, and ovaries, among others [37].

### 3.4. Treatment of LPS-Stimulated RAW 264.7 Cells with *Kigelia africana* Extracts Inhibits Nitric Oxide (NO) Production

The suppression effect of the extracts on NO production is presented in Table 3. Nitric oxide is a toxic free radical produced by macrophages in response to LPS via oxidation of the terminal guanidine nitrogen atom from L-arginine by nitric oxide synthase (NOS) [38]. Inducible NOS is involved in overproduction of NO in response to proinflammatory mediators such as IL-1F3, TNF-*α*, and bacterial LPS [39]. While a small amount of NO synthesized by endothelial NOS (eNOS) and neuronal NOS (nNOS) is essential for maintaining homeostasis, a significantly increased amount of NO synthesized by iNOS participates in provoking inflammatory process and acts synergistically with other inflammatory mediators. Hence, to control its production is a principal focus in an anti-inflammatory investigation. In this study, the extracts had a concentration-dependent inhibition of NO production. At the lowest concentration (1.6 *µ*g/mL), KFM02 released the lowest amount of NO (35.35 ± 14.86%), therefore having the best NO inhibition capacity and highest cell viability (113.21 ± 16.26%). Likewise, at the highest concentration (100 *µ*g/mL), KFM02 released the lowest amount of NO (54.83 ± 8.18%), having the best NO inhibition capacity and highest cell viability (99.88 ± 3.57%). All the extracts compared well with the standard (quercetin) at both low (64.17 ± 7.01% inhibition/97.53 ± 5.03% cell viability) and high concentrations (94.37 ± 3.31% inhibition/57.05 ± 7.66% cell viability). A slight reduction in cell viability was noticeable as the concentration increased, yet it was clear that even at the highest concentration (100 *μ*g/mL), the percentage of cell viability for all the tested extracts remained above 90%, inferring a noncytotoxic concentration. Therefore, among the extracts, KFM02 was the best NO inhibitor with high cell viability. Nonetheless, all the extracts had a strong ability to inhibit NO production while leaving normal mammalian cells unaffected. The inhibition of NO production by plant extracts may be due to the inhibition of iNOS expression [40]. The extracts in our study had good inhibitory activity on NO production and a low cytotoxicity; therefore, they could be used to mitigate the propagation of inflammation by NO. The reduction of NO is also associated with the reduction of oxidative stress [41].

### 3.5. *Kigelia africana* Extracts Strongly Suppress Cyclooxygenase-2 (COX-2) Enzyme

**Figure 1:**
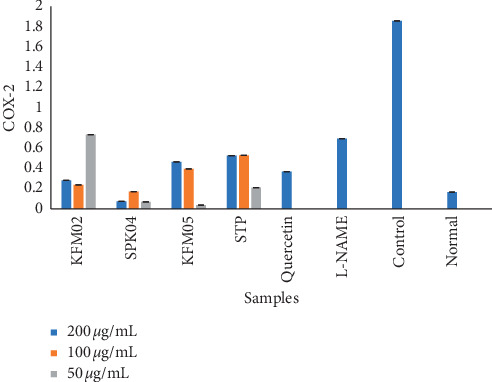
Inhibition of cyclooxygenase-2 enzyme activity by *K. africana* aqueous, methanol, and acetone extracts.

COX-2 is an inducible enzyme which causes the production of PGs and is closely associated with acute and chronic inflammatory disorders [42]. Prostaglandins produced by COX-2 play an important role in the physiological functions of some tissues [43]. Despite some positive features, expression of COX-2 is high in LPS-stimulated inflammation-related cell types (macrophages and mast cells) [44]. The results of the COX-2 study are presented in Figure 1. At the highest concentration (200 *µ*g/mL), SPK04 was the best COX-2 inhibitor, followed by KFM02 and KFM05. At 200 *µ*g/mL, SPK04 and KFM02 were better COX-2 inhibitors than quercetin, the untreated control. At 100 *µ*g/mL, SPK04 was the best COX-2 inhibitor, followed by KFM02 and KFM05. At the same concentration, SPK04 and KFM02 were also better COX-2 inhibitors than quercetin and compared well with the untreated control. At the lowest concentration of 50 *µ*g/mL, KFM05 was the best COX-2 inhibitor, followed by SPK04 and KFM02. At this concentration, KFM05 and SPK04 were better inhibitors than quercetin. At 50 *µ*g/mL, KFM02 had the same COX-2 inhibition capacity as L-NAME, which is an inhibitor of NO synthesis. Overall, SPK04 was the best COX-2 inhibitor, followed by KFM05 and KFM02. Our study results showed that the tested plant extracts had higher COX-2 inhibitory effect at the same concentration as quercetin. The COX-2 inhibitory capacity of the plant extracts being better than that of the standard is evidence of the potency of these extracts in inhibiting COX-2 enzyme activity. The COX-2 inhibitory capacity of these extracts may be explained by the presence of tannins [45]. Tannins affect highly purified enzyme-based targets due to their ability to bind strongly with proteins and this may lead to the inhibition of PG synthesis through blocking the COX enzymes. For anti-inflammatory herbal extracts or herbal-derived compounds, inhibition of COX-2 has commonly been found to be their molecular target [44].

Eldeen and Van Staden [46] reported the COX-1 and COX-2 inhibitory effect of *K africana* leaf ethanol and bark dichloromethane extracts. Whereas not much has been reported on the COX-2 inhibitory effect of *K. africana*, other studies have reported the inhibitory effect of COX by medicinal plants. Seven lignans and one dihydrochalcone isolated from the leaves of *Pleurothyrium cinereum* and *Ocotea macrophylla* were found to be potent inhibitors of COX-2 [47]. Ethyl acetate-soluble extract of the stems of *Macrococculus pomiferus* was found to inhibit COX-2 [48]. (S)-Coriolic acid and (±)-glycerol 1-monolinolate isolated from ethyl acetate-soluble extract of the seeds of *Hernandia ovigera* showed selective inhibitory activity with cyclooxygenase-2 [49]. The compound 2,4,5-trimethoxybenzaldehyde isolated from *Daucus carota* seed extracts inhibited COX-2 enzyme very significantly at a concentration of 100 *μ*g/mL [50]. These studies are evidence of the anti-inflammatory activity of plant extracts through COX inhibition although more research needs to be conducted regarding the COX-2 inhibitory effect of all *K. africana* plant extracts including their mechanisms of action.

### 3.6. *Kigelia africana* Extracts Suppress Expression of Proinflammatory Cytokines and Have No Effect on Anti-Inflammatory Cytokine Expression

Cytokines are extremely important for the maintenance of optimum inner environment of our system and for protection from injurious agents. However, they should be regulated and kept at favourable levels. Accumulation of a high level of proinflammatory cytokines amplifies inflammation due to the interdependency of one on the other as the production of proinflammatory cytokines works in a cascade mechanism by downstream effects of earlier released cytokines [51]. For example, IL-1 stimulates TNF-*α* and IL-6 [52]; meanwhile, the prostaglandin-E2 (PGE2) promotes the release of IL-6. Therefore, suppressing the production of a cytokine may influence the reduction of another dependent cytokine. The major proinflammatory cytokines that are responsible for early responses, namely, IL-1*α*, IL-1*β*, IL-6, and TNF-*α*, are elevated in LPS-induced macrophages. Interleukin-6 (IL-6), interleukin-1*β* (IL-1*β*), and TNF-*α* participate in the prolonging of chronic inflammation. Therefore, these cellular messengers should be mitigated as a measure to avoid further inflammatory process. Interleukin-10 is an anti-inflammatory cytokine which plays a central role in limiting host immune response to pathogens, thereby preventing damage to the host and maintaining normal tissue homeostasis. Unlike proinflammatory cytokines, IL-10 cellular messengers should be promoted to avoid further inflammatory process.

**Figure 2:**
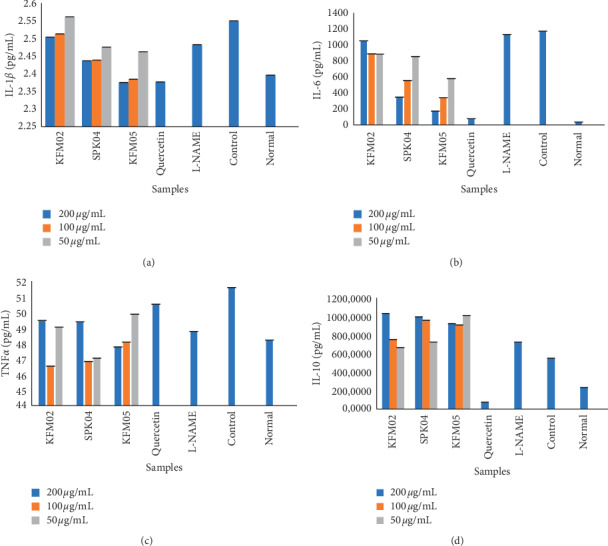
The effect of *K. africana* aqueous (KFM02), methanol (SPK04), and acetone (KFM05) extracts on interleukin-6 (IL-6), IL-1*β*, tumor necrosis factor-*α* (TNF-*α*), and IL-10 in LPS-stimulated RAW 264.7 macrophage cells. The values of cytokine production were expressed in pg/mL, and the data presented were the mean values of three experiments ±SEM. *p* < 0.05, the LPS-treated group versus the control group. Quercetin was used as the standard; the control was made of untreated LPS-stimulated RAW 264.7 macrophage cells, and L-NAME (N omega-Nitro-L-arginine methyl ester hydrochloride) is a nitric oxide synthase inhibitor treated with the extracts. (a) Interleukin-1*β* (IL-1*β*). (b) Interleukin-6 (IL-6). (c) Tumor necrosis factor-*α* (TNF-*α*). (d) Interleukin-10 (IL-10).

In this study, exposure of the cells with LPS significantly increased the production of IL-6, IL-1*β*, and TNF-*α*. However, treatment with *Kigelia* extracts suppressed the production of the cytokines in some cases, and in others, there was no suppression (Figure 2). At the highest concentration (200 *µ*g/mL), KFM05 expressed a strong inhibitory effect on IL1-*β* which was similar to that of the standard (quercetin). This was followed by SPK04, and KFM02 had the least inhibitory effect although it was higher than that of the untreated control and not significantly different from L-NAME inhibition capacity. At the lowest concentration (50 *µ*g/mL), KFM05 still had the best IL-1*β* inhibitory effect although it was much lower than the standard but not significantly different from SPK04. At this concentration, KFM02 had no inhibitory effect since it was not significantly different from the control where the cells were stimulated but not treated. Therefore, at all concentrations (200, 100, and 50 *µ*g/mL), KFM05 was the best IL-1*β* inhibitor, followed by SPK04 and KFM02. At 200 and 100 *µ*g/mL, the inhibitory effect of KFM05 was not significantly different from the standard, making it an excellent IL1-*β* inhibitor.

KFM05 had the highest IL-6 inhibition capacity at 200 *µ*g/mL followed by SPK04 while KFM02 had the least inhibitory effect which was not significantly different from the untreated control. Implying that KFM02 did not inhibit cytokine IL-6 at 200 *µ*g/mL. At 100 *µ*g/mL, KFM05 was the best IL-6 inhibitor followed by SPK04 and very little inhibitory effect was observed for KFM02. At the lowest concentration (50 *µ*g/mL), KFM05 had the highest IL-6 inhibitory effect. Extracts SPK04 and KFM02 had fairly good inhibition effect and there was no significant difference in IL-6 inhibitory effect by these two extracts at 50 *µ*g/mL. KFM05 was the best IL-6 inhibitor at all concentrations, followed by SPK04, and KFM02 had the least inhibition. At 100 and 50 *µ*g/mL, KFM02 had the same IL-6 inhibition capacity. There was no significant difference between L-NAME inhibitory effect and the untreated control. This implies that L-NAME had barely any inhibitory effect on IL-6. The results are presented in Figure 2.

KFM05 had the highest TNF-*α* inhibition effect at 200 *µ*g/mL (Figure 2). At the same concentration, the inhibitory effect of SPK04 and KFM02 was not significantly different. At 100 *µ*g/mL, KFM02 had the highest TNF-*α* inhibitory effect followed by SPK04 and KFM05. The inhibitory effects of KFM05 at 200 and 100 *µ*g/mL were not significantly different to those of SPK04. At the lowest concentration (50 *µ*g/mL), SPK04 had the highest TNF-*α* inhibition effect followed by KFM02, and KFM05 had the least inhibition effect. Generally, at 100 *µ*g/mL, all the three extracts had the highest inhibition capacity for TNF-*α*. Furthermore, at all concentrations, the three plant extracts had a higher inhibitory effect for TNF-*α* than the standard (quercetin) and the untreated control. L-NAME had an inhibitory effect on TNF-*α*. Therefore, the extracts investigated could inhibit the expression of IL-6, IL-1*β*, and TNF-*α* molecules which have been demonstrated as the target for many anti-inflammatory medicinal plants and their compounds [7].

Interleukin-10 is an anti-inflammatory cytokine structurally related to interferons [53] that plays a central role in limiting the immune response to pathogens, preventing damage to the host, and maintaining normal tissue homeostasis. All the tested extracts had no effect on the anti-inflammatory cytokine IL-10 (Figure 2). L-NAME had no inhibitory effect on IL-10 while the standard greatly inhibited IL-10.

The good antioxidant and anti-inflammatory activity of *K. africana* fruit extracts may be explained by the presence of verminoside [54], an iridoid glycoside. *In vitro* assays have confirmed that verminoside has significant anti-inflammatory effects, inhibiting both iNOS expression and NO release in the LPS-induced J774.A1 macrophage cell line [55]. Using the carrageenan-induced paw edema model in rats, as well as the acetic acid-induced writhing, hot plate, and formalin-induced paw licking models in mice, Carey et al. [56] reported that the flower extract of *K. africana* exhibited significant anti-inflammatory and analgesic activities at doses of 100, 200, and 400 mg/kg body weight in rats and mice. Furthermore, the methanolic extract of *K. africana* fruit using various *in vivo* models of inflammation in mice and rats significantly attenuated the inflammation in formaldehyde-induced paw edema, acetic acid-induced vascular permeability, cotton pellet-induced granuloma, estimation of plasma MDA levels, and carrageenan-induced peritonitis models [57]. Verminoside also exhibits cytotoxic activity in the concentration range of 70–355 *µ*M [54]. Hassan et al. [58] reported that *K. africana* extracts have significant wound-healing properties observing a rapid reduction in exudation and scab formation, classical signs of inflammation. The same study also reported that the lethal dose of the leaf extract is greater than 3 g/kg; this suggests that *K. africana* is safe to be consumed for the treatment of various ailments. It is proposed that the mechanism of action of *K. africana* leaf extracts may be due to its angiogenic and mitogenic potential leading to increased cellular proliferation and collagen synthesis [58], thereby assisting in scar tissue formation and reperfusion of the injured site, thus resolving the sequela of inflammation.

Among the three tested plant extracts, the polymethanolic extract (SPK04) showed more potency in all experiments as compared to the mono extracts KFM05 and KFM02. SPK04 is a combination of *K. africana* leaves and *S. campanulata* leaves in equal amounts. This extract was made to mimic the traditional preparation of these plants before their usage in wound healing and skin infections [17]. Herbal medicine preparations are made of either a single plant or a combination of more than one plant. When two or more plants are used to make a herbal preparation, the resultant is a polyherbal formulation (PHF). Polyherbalism is more common than single herbal preparations and this also dates to ancient times [59]. The therapeutic effect of a polyherbal is based on the pharmacokinetic and pharmacodynamic synergism of the different herbs in the preparation [60]. Therefore, since in polyherbals a combination of phytochemicals acts on multiple targets at the same time, therapeutic activity is more significant than that of a single herb preparation [61] which may explain the potency of SPK04. The extracting solvents used in this study were water, acetone, and methanol. The methanolic extract (SPK04) showed more potency than the hot water (KFM02) and acetone (KFM05) extracts. The potency of the methanolic extract may be explained by the ability of methanol to extract lower molecular weight polyphenols, whereas water and acetone are good for the extraction of higher molecular weight flavanols [62]. Furthermore, it has been reported that the free radical scavenging activity of *K. africana* may be due to the presence of phenols that can donate the hydrogen atoms in their hydroxyl groups [27]. Thus, the potency of SPK04 may be explained by its polyherbal nature and the nature of the extraction solvent used.

## 4. Conclusion

The *in vitro* antioxidant assays indicate that the tested plant extracts are significant sources of natural antioxidants, which might be helpful in preventing oxidative stress. Additionally, there is a potential that these extracts could be used as additives in the food industry for purposes of providing good protection against oxidative damage. Our study results also indicated that the extracts exhibited anti-inflammatory properties by suppressing the expression of proinflammatory cytokines (IL-1 *β*, IL-6, and TNF-*α*) in LPS-stimulated macrophages and COX-2 enzyme; thus, the hypothesis of the study was accepted. Fortunately, our extracts did not suppress the anti-inflammatory cytokine (IL-10) which further justifies their selective inhibitory activity on important cytokines. Lastly, results of this study suggest that the bioactive constituents of *K. africana* extracts and *S. campanulata* in SPK04 had both antioxidant and anti-inflammatory activities. Antioxidants act by scavenging free radicals such as reactive oxygen species, hydroxyl radicals, and nitric oxide while anti-inflammatory mediators act by modulating the activities of proinflammatory enzymes and cytokines. The study also gives insights on *K. africana*'s safety to normal mammalian cells. Thus, we recommend further research be conducted to isolate, identify, and characterize the bioactive compounds that are responsible for the activities. Once the active compounds have been isolated, the mechanism of activity can be examined. Prior to clinical use, the *in vivo* antioxidant and anti-inflammatory activity of these extracts need to be assessed.

## Figures and Tables

**Table 1:** Percentage ABTS radical scavenging activity of *Kigelia africana* extracts.

Concentrations (mg/mL)	ABTS radical scavenging activity (mean % ± SEM (*n* *=* 3)			
	SPK04	KFM02	KFM05	Ascorbic acid^*∗∗∗*^
100.00	99.37 ± 0.56	109.12 ± 14.32	100.33 ± 1.11	103.95 ± 4.09
50.00	99.10 ± 0.89	85.34 ± 14.35	99.70 ± 0.52	104.63 ± 4.64
25.00	83.7 ± 21.01	47.48 ± 18.09	81.52 ± 16.20	99.52 ± 0.88
12.50	74.94 ± 3.68	29.77 ± 16.76	51.39 ± 13.55	100.09 ± 0.22
6.25	45.83 ± 8.75	27.14 ± 8.94	31.21 ± 13.55	95.30 ± 4.92
3.13	35.82 ± 0.00	19.75 ± 6.85	20.35 ± 2.96	99.73 ± 0.27
1.56	18.03 ± 4.25	15.3 ± 0.88	13.92 ± 13.30	99.97 ± 0.12

^*∗∗∗*^Positive control.

**Table 2:** Antioxidant, 15-LOX inhibition activity, and cytotoxicity of *Kigelia africana* extracts.

Samples	IC_50_ values (*μ*g/mL)		LC_50_ values (*μ*g/mL)
	ABTS assay	15-LOX inhibition	Cytotoxicity on Vero cells
SPK04	4.28	>1000	0.32 ± 0.11
KFM02	21.29	307.12 ± 4.68	0.20 ± 0.01
KFM05	19.47	>1000	0.26 ± 0.12
Ascorbic acid^*∗∗∗*^	−0.02 ± 0.00	ND	ND
Quercetin^*∗∗∗*^	ND	24.69 ± 1.43	ND
Doxorubicin^*∗∗∗*^	ND	ND	0.01 ± 0.00

^*∗∗∗*^Positive control; ND: not detected. Values are expressed as mean ± SEM.

**Table 3:** Inhibition of nitric oxide (NO) production by *Kigelia africana* extracts.

Samples	Concentration (*μ*g/mL)	% NO inhibition	% cell viability
KFM02	1.60	35.35 ± 14.86	113.21 ± 16.26
	12.50	46.55 ± 14.71	96.99 ± 6.16
	50.00	55.82 ± 12.00	94.95 ± 2.86
	100.00	54.83 ± 8.18	99.88 ± 3.57
SPK04	1.60	42.23 ± 16.07	112.79 ± 15.01
	12.50	44.87 ± 15.74	101.72 ± 10.90
	50.00	50.40 ± 10.48	97.65 ± 9.97
	100.00	59.65 ± 4.05	97.75 ± 10.96
KFM05	1.60	37.16 ± 18.86	104.95 ± 12.93
	12.50	54.65 ± 9.83	100.39 ± 5.87
	50.00	59.67 ± 3.50	99.41 ± 0.73
	100.00	63.43 ± 6.08	95.34 ± 4.14
Quercetin	1.60	64.17 ± 7.01	97.53 ± 5.03
	12.50	90.57 ± 3.94	83.52 ± 3.87
	50.00	91.05 ± 5.99	61.31 ± 2.19
	100.00	94.37 ± 3.31	57.05 ± 7.66
Doxorubicin	2.00		89.17 ± 5.65
	4.00		60.22 ± 5.19
	10.00		15.55 ± 6.72
	20.00		4.23 ± 4.06

Values are expressed as mean ± SEM.

## Data Availability

All data supporting our findings are adequately included within the article.

## Abbreviations

DPPH: 2,2-Diphenyl-1-picrylhydrazyl
ABTS: 2,2′-Azino-bis (3-ethylbenzthiazoline-6-sulfonic acid
MTT: 3-(4,5-Dimethylthiazol-2-yl)-2,5-diphenyl tetrazolium bromide
LPS: Lipopolysaccharides
ROS: Reactive oxygen species
OS: Oxidative stress
NO: Nitric oxide
PGs: Prostaglandins
IL: Interleukin
TNF-*α*: Tumor necrosis factor-alpha
ANOVA: Analysis of variance
COX-2: Cyclooxygenase-2
15-LOX: 15-Lipoxygenase
PUFAs: Polyunsaturated fatty acids
DMSO: Dimethylsulphoxide
NSAIDs: Nonsteroidal anti-inflammatory drugs
SOD: Superoxide dismutase
PBS: Phosphate-buffered saline
DMEM: Dulbecco's modified Eagle's medium
L-NAME: N-Omega-nitro-L-arginine methyl ester hydrochloride.
